# Structural characteristics of gut microbiota in longevity from Changshou town, Hubei, China

**DOI:** 10.1007/s00253-024-13140-3

**Published:** 2024-04-15

**Authors:** Xu Ai, Yu Liu, Jinrong Shi, Xiongwei Xie, Linzi Li, Rui Duan, Yongling Lv, Kai Xiong, Yuanxin Miao, Yonglian Zhang

**Affiliations:** 1 Jingmen Central Hospital, Hubei Clinical Medical Research Center for Functional Colorectal Diseases, Jingmen, 448000 Hubei China; 2 Maintainbiotech. Ltd. (Wuhan), Wuhan, 430000 Hubei China; 3 Research Institute of Agricultural Biotechnology, Jingchu University of Technology, Jingmen, 448000 Hubei China

**Keywords:** Gut microbiota, Longevity, Microbial diversity, *Akkermansia*

## Abstract

**Abstract:**

The gut microbiota (GM) and its potential functions play a crucial role in maintaining host health and longevity. The aim of this study was to investigate the potential relationship between GM and longevity. We collected fecal samples from 92 healthy volunteers (middle-aged and elderly: 43–79 years old; longevity: ≥ 90 years old) from Changshou Town, Zhongxiang City, Hubei, China. In addition, we collected samples from 30 healthy middle-aged and elderly controls (aged 51–70 years) from Wuhan, Hubei. The 16S rDNA V3 + V4 region of the fecal samples was sequenced using high-throughput sequencing technology. Diversity analysis results showed that the elderly group with longevity and the elderly group with low body mass index (BMI) exhibited higher α diversity. However, no significant difference was observed in β diversity. The results of the microbiome composition indicate that *Firmicutes*, *Proteobacteria*, and *Bacteroidota* are the core phyla in all groups. Compared to younger elderly individuals, *Akkermansia* and *Lactobacillus* are significantly enriched in the long-lived elderly group, while *Megamonas* is significantly reduced. In addition, a high abundance of *Akkermansia* is a significant characteristic of elderly populations with low BMI values. Furthermore, the functional prediction results showed that the elderly longevity group had higher abilities in short-chain fatty acid metabolism, amino acid metabolism, and xenobiotic biodegradation. Taken together, our study provides characteristic information on GM in the long-lived elderly population in Changshou Town. This study can serve as a valuable addition to the current research on age-related GM.

**Key points:**

*• The gut microbiota of elderly individuals with longevity and low BMI exhibit higher alpha diversity*

*• Gut microbiota diversity did not differ significantly between genders in the elderly population*

*• Several potentially beneficial bacteria (e.g., Akkermansia and Lactobacillus) are enriched in long-lived individuals*

**Supplementary Information:**

The online version contains supplementary material available at 10.1007/s00253-024-13140-3.

## Introduction

The social problems brought about by population aging are increasingly evident. How to delay aging, alleviate, and maintain the health of the elderly has become the focus of the times (Chedraui and Pérez-López 2013; Bao et al. 2022; Wang et al. 2022). Although thanks to today’s medical technology, human life expectancy has increased, the maximum human lifespan seems to be limited by natural conditions (Dong et al. 2016; Caruso et al. 2022). It is well known that aging is closely related to various factors such as genetics and the environment. The Human Genome Project (HMP) has confirmed the existence of numerous genes associated with lifespan extension. However, it appears that genetic factors only account for 20–40% of the overall influence on lifespan, with the majority being attributed to environmental factors (Finlay et al. 2019; van den Berg et al. 2019; Wu et al. 2021a). With the implementation of the HMP, scientists studying longevity and aging have begun to link longevity genes with the microbiome. The gut microbiota (GM), which is related to the development of various diseases, has become a hot topic in this field. Recent review summarizes the important role played by GM in the aging process (Shi et al. 2022; Li and Roy 2023). However, the biological mechanisms of aging are complex and unclear. Therefore, further research on the GM is still needed.

Research on the longevity of various model organisms has shown that the GM is involved in regulating the host’s lifespan. In the pursuit of understanding the mechanisms of longevity, organisms that are easy to breed and have a shorter lifespan have emerged as the most suitable choice. *Drosophila*, as a classic model organism, is frequently utilized in genetic research. The research results indicate that metabolites derived from GM may activate the host’s immune system, thereby influencing the proliferation of intestinal stem cells and lifespan (Fan et al. 2018; Onuma et al. 2023). *Caenorhabditis elegans* is a simple organism that feeds on bacteria and reproduces rapidly. It is widely used in various disease models and studies on microbiota-related mechanisms. Gomez et al.’s (2012) research shows that specific probiotic strains can influence the lifespan of *C. elegans* by preventing intestinal infections. The finding has also been validated in mouse models (Matsumoto et al. 2011). Blind subterranean mole-rats and naked mole rats, both long-lived rodents, have shown unique compositions of GM that are associated with longevity (Debebe et al. 2017; Sibai et al. 2020). Interestingly, GM transplantation experiments in fish have also confirmed the key role of GM in regulating host lifespan (Smith et al. 2017).

Life is the greatest creation of nature. During the process of childbirth, babies acquire microbiota from their mothers, but throughout the course of life from growth and development to aging and death, this microbiota acquired at birth can rarely persist (Ku et al. 2020; Santaella-Pascual et al. 2023; Walker and Hoyles 2023). The GM is a complex functional ecosystem that plays a crucial role in non-genetic factors that affect individual health and lifespan, accompanying the host from birth to death. There have been numerous studies on the association of GM with various age-related diseases, and disruptions in the GM may lead to cardiovascular and cerebrovascular diseases, cancer, and other metabolic system-related diseases, thereby affecting the health and lifespan of the host (Hirata et al. 2020; Son and Kim 2022; Son and Cho 2023). Long-lived individuals have a lower incidence of inflammatory-related diseases and other potential infection-related diseases. This is associated with the presence of certain microorganisms in their intestines that can produce anti-inflammatory and antioxidant activities (Park et al. 2015a; Sato et al. 2021). A study on the GM of long-lived individuals in Italy showed differences in the core gut bacteria among different age groups of elderly individuals (Biagi et al. 2016). Furthermore, a functional analysis of the GM of centenarians from Sardinia, Italy, indicated that they have a higher metabolic capacity for short-chain fatty acids (SCFA) (Wu et al. 2019). A large Mendelian randomization analysis by Gagnon et al. (2023) showed that *Prevotella* and *Paraprevotella* are enriched in long-lived individuals, but this result has not been confirmed in European populations. In addition, the enrichment of *Akkermansia*, *Bifidobacterium*, and *Christensenellaceae* in long-lived individuals may support healthy aging (Biagi et al. 2016; Badal et al. 2020).

Longevity, as one of the most complex phenotypes, has intricate and unclear biological mechanisms and is closely associated with GM (Li and Roy 2023; Miller et al. 2023; Coradduzza et al. 2023; Rahman et al. 2023). Although omics technologies have advanced the research on GM and longevity, there is still a lack of substantial data support in this field to further validate the microbiota biomarkers associated with longevity (Coradduzza et al. 2023; Lee et al. 2023; Miller et al. 2023; Qiao et al. 2023). Additionally, there are regional differences in GM among long-lived populations. Therefore, it is particularly important to study the GM of long-lived elderly populations in different regions, which can not only improve the existing GM databases of long-lived populations but also help us decipher the unique microbiota composition of regional long-lived populations and further explore potential microbial factors related to longevity. Zhongxiang, Hubei, is a famous longevity town in China, but the GM characteristics related to longevity in this region have not been fully studied. In order to clarify the unique GM features of long-lived elderly individuals in this region, this study selected the elderly population in Changshou Town as the research subjects, including long-lived individuals, their descendants living with them, and neighbors of the same age range from non-long-lived families, with the control group being local healthy aborigines in Wuhan City. We obtained the GM composition and predicted potential functions of the elderly population living in Changshou Town and compared them with healthy elderly individuals in nearby Wuhan City. In addition, we also conducted correlation analysis between GM features and BMI (body mass index). This study comprehensively analyzes the characteristics of GM among the elderly in the town of longevity and contributes to further research on GM in healthy aging.

## Material and methods

### Volunteer recruitment and experimental design

The volunteers recruited for this study are from Changshou Town, Zhongxiang, Hubei, China. Information such as age, gender, height, weight, medical history, and dietary habits were obtained through questionnaires. Volunteers with a history of major diseases, chronic illnesses, excessive smoking and drinking, dietary preferences, and recent medical care or antibiotic treatment within the past 3 months were excluded. Ultimately, 33 healthy elderly individuals (above 90 years old) and their cohabiting offspring (34 individuals), as well as 25 non-long-lived neighbors of similar age to the offspring group (parents as non-long-lived elderly), were recruited. Additionally, fecal samples from 30 healthy volunteers from non-long-lived families in a nearby city (Wuhan) were selected as the control group. This study has been approved by the Ethics Committee of Jingmen Central Hospital (Approval No: [202302229]), and all participants have signed informed consent forms. The 122 recruited healthy volunteers were divided into four groups: Longevity Group (LG) — 33 long-lived participants; Offspring Group (OG) — 34 offspring of long-lived participants; Neighbor Group (NG) — 25 elderly individuals from non-long-lived families in Changshou Town; Control Group (CG) — 30 elderly individuals from non-long-lived families in Wuhan City. In order to investigate the potential impact of gender differences on age-related GM, the four groups were further divided into eight subgroups based on gender (Table 1). In addition, we found that the long-lived elderly group seemed to have lower BMI values, so we grouped all volunteers according to their BMI values as follows: low BMI group (LB group): (*n* = 18, BMI ≤ 19), medium BMI group (MB group): (*n* = 79, 19 < BMI ≤ 25), and high BMI group (HB group): (*n* = 25, BMI > 25) (Table 2).

**Table 1:** Demographic characteristics of volunteers

Parameters	Research group				Control group			
	LG (*n* = 33)		OG (*n* = 34)		NG (*n* = 25)		CG (*n* = 30)	
Male/Female	LGM (*n* = 18)	LGF (*n* = 15)	OGM (*n* = 23)	OGF (*n* = 11)	NGM (*n* = 11)	NGF (*n* = 14)	CGM (*n* = 15)	CGF (*n* = 15)
Age	92.61 ± 2.59	93.13 ± 3.2	63.09 ± 8.11	61.27 ± 7.18	59.09 ± 5.72	56.14 ± 8.65	59.07 ± 5.52	60.47 ± 5.79
Height (m)	1.62 ± 0.07	1.53 ± 0.09	1.69 ± 0.05	1.63 ± 0.07	1.65 ± 0.07	1.62 ± 0.08	1.72 ± 0.11	1.6 ± 0.04
Weight (kg)	53.11 ± 7.93	46.22 ± 9.96	65.44 ± 0.24	59.44 ± 6.89	60.89 ± 7.26	61.22 ± 8.86	72.08 ± 1.06	56.41 ± 7.21
BMI (kg/m^2^)	20.28 ± 2.54	19.61 ± 2.88	22.96 ± 2.97	22.46 ± 2.19	22.43 ± 2.33	23.97 ± 2.8	24.31 ± 1.7	22.65 ± 3.09

LGM, longevity group male; LGF, longevity group female; OGM, offspring group male; OGF, offspring group female; NGM, neighbor group male; NGF, neighbor group female; CGM, control group male; CGF, control group female

**Table 2:** BMI grouping information

	LB (*n* = 18, BMI ≤ 19)	MB (*n* = 79, 19 < BMI ≤ 25)	HB (25, BMI > 25)
BMI (kg/m^2^)	17.43 ± 1.61	22.12 ± 1.75	26.28 ± 0.8
Age	86.44 ± 13.25	70.06 ± 17.21	59.22 ± 6.98
Male/Female	8/10	45/34	14/11
Height (m)	1.56 ± 0.07	1.62 ± 0.08	1.64 ± 0.08
Weight (kg)	42.61 ± 6.11	52.19 ± 4.99	69.7 ± 7.43

All values are presented as mean ± SD

### Sample collection and DNA extraction

Stool samples were collected by the participants themselves or their family members using a stool collection kit. 1–2 g of stool was then placed in a stool sample collection tube and immediately stored in a − 20 °C freezer. The samples were collected by the researchers within a week and stored at − 80 °C. Stool sample DNA extraction was performed using the HiPure Stool DNA Mini Kit (Magen, Guangzhou, China).

### PCR amplification and 16S rRNA gene sequencing

The universal primers (341F: 5′-CCTACGGGNGGCWGCAG-3′ and 805R: 5′-GACTACHVGGGTATCTAATCC-3′) targeting the V3-V4 hypervariable region of the 16S rRNA gene were used for PCR amplification. The PCR conditions were as follows: 95 °C for 3 min, followed by 25 cycles of 95 °C for 30 s, 55 °C for 30 s, and 72 °C for 15 s, with a final extension at 72 °C for 5 min and storage at 4 °C. The concentration of the PCR product purified with AMPure XT beads (Beckman Coulter Genomics, Danvers, MA, USA) was measured using Qubit Fluorometer (Invitrogen, Carlsbad, CA, USA), and the product was finally checked by 1.5% agarose gel electrophoresis. The mixed PCR products were used for library construction, with sequencing adapters added and library index information recorded. The reaction conditions were as follows: pre-denaturation at 98 °C for 45 s, followed by 8 cycles of 98 °C for 15 s, 60 °C for 30 s, and 72 °C for 30 s, with a final extension at 72 °C for 10 min and storage at 4 °C. After PCR amplification, AMPure XP beads (Beckman, Brea, CA, USA) were used to remove primer dimers and small fragments. Prior to sequencing, the library concentration was quantified and calculated using Qubit. The validated library was sequenced on the Illumina Miseq platform, generating 2 × 250 bp paired-end reads.

### Bioinformatics analysis and statistical analysis

Trimming of the primers from the raw paired-end sequences was performed using the QIIME2 platform (Bolyen et al. 2019). The default parameters of DADA2 (version 1.29.0) were used for subsequent quality filtering, merging, dereplication, denoising, and chimera removal (Callahan et al. 2016). This process resulted in generating an output file that contains amplicon sequence variants (ASVs). The ASVs were then mapped to the Silva reference database (version 138) for taxonomic classification annotation (Quast et al. 2013). The evaluation of α-diversity indices, which include community richness, community diversity, and community evenness, was performed using QIIME2. The evaluation of β-diversity, which demonstrates differences in microbial community composition between different groups, was conducted using principal coordinate analysis (PCoA) with the Bray–Curtis, Abund-Jaccard, Unweighted Unifrac, and Weighted Unifrac algorithms. The default parameters of the PICRUSt2 software (Douglas et al. 2020) were used to generate predicted genomes based on the ASV file and to explore potential gene functions in the GM using the KEGG database (Kanehisa et al. 2023) for comparison. The comparison of potential gene functions among groups was performed using the Kruskal–Wallis test. Linear discriminant analysis effect size (LEfSe) was used to identify bacterial taxa that were significantly different (log LDA score > 2 and *p* < 0.05). R version 4.2.1 (R Foundation for Statistical Computing, Vienna, Austria) was used for visual analysis. All values are presented as mean ± standard deviation (SD). *p* < 0.05 indicates statistical significance.

## Results

### Gut microbiota diversity

**Fig. 1:**
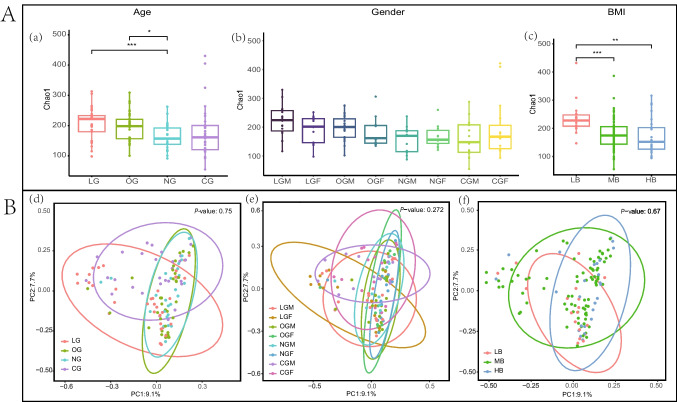
Venn diagrams illustrate the shared and unique ASVs among four age groups (**A**), eight gender groups (**B**), and three BMI groups (**C**). Histograms display phyla with a mean relative abundance exceeding 0.01% across all samples (**D**) and genera with a mean relative abundance exceeding 1% (**E**)

We obtained 10,423,099 raw reads and 6,417,699 high-quality 16S rRNA gene sequences from 122 fecal samples, with an average of 52,604 sequences per sample. A total of 4317 bacterial ASVs were identified, with each sample containing 157–406 ASVs (Supplementary Table S1). These ASVs are taxonomically annotated into 15 phyla, 19 classes, 54 orders, 92 families, and 284 genera. Venn diagram-based analysis showed that 497 core ASVs were shared between the LG and the other three groups. Among them, the LG had 896 unique ASVs and shared the highest number of ASVs (972) with the OG. Further grouping by gender revealed that there were 208 core ASVs shared among the eight groups. The commonly observed ASVs among the three groups based on BMI accounted for 19.2%. It is worth noting that as the BMI value increases, the number of unique ASVs exhibited a decreasing trend (Fig. 1A). The relative abundance bar charts at the phylum and genus levels were plotted based on ASV species annotation information (Fig. 1B). The dominant phyla with an average relative abundance > 10% in all samples were *Firmicutes* (61.8 ± 23.5%), *Proteobacteria* (19.2 ± 23.4%), and *Bacteroidota* (12.9 ± 17.9%). The dominant genera with an average relative abundance > 5% were *Escherichia-Shigella* (8.8 ± 17.4%), *Prevotella* (6.3 ± 14.6%), *Bacteroides* (6.0 ± 11.3%), *Faecalibacterium* (5.8 ± 7.5%), and *Subdoligranulum* (5.5 ± 8.3%).

### Alpha and beta diversity of gut microbiota

**Fig. 2:**
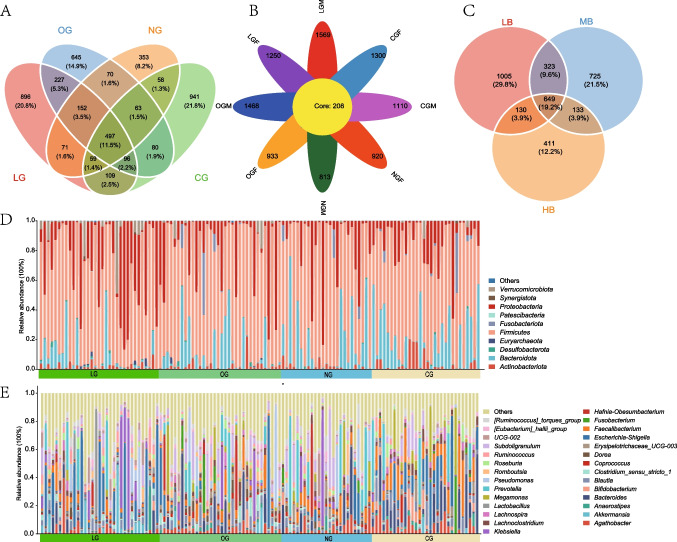
(**A**) Alpha diversity results based on the Chao1 index. Box plots show the Chao1 index for four age groups (a), eight gender groups (b), and three BMI groups (c). (**B**) Beta diversity results based on the Bray–Curtis distance algorithm. (d–f) Showed the differences in the overall structure of inter-group microbial communities under three different grouping patterns. *, p < 0.05; **, p < 0.01; ***, p < 0.001

The richness and evenness of the GM community are evaluated using α diversity indices, including the Chao1, Shannon, and Simpson indices. Taking the Chao1 index as an example, in the age group pattern, LG and OG show significantly higher values compared to NG (Fig. 2A). However, there is no significant difference in the Chao1 index between LG and OG, as well as between LG and CG. Nevertheless, we can still see that the Chao1 index of LG is relatively higher compared to the other three groups, indicating a higher alpha diversity. In the gender group analysis, the alpha diversity results based on the Chao1 index indicate no significant differences between any two groups. Interestingly, in the BMI-based group analysis, we observed that the α diversity of the elderly group with low BMI values is significantly higher than that of the other two groups. Beta diversity is assessed using four algorithms: Bray–Curtis, Abund-Jaccard, Unweighted-UniFrac, and Weighted-UniFrac. Results using the Bray–Curtis method, for example, show no significant differences among the three grouping patterns (*p* > 0.05). Nevertheless, we can still observe a high consistency in the community composition structure between the OG and NG groups, while there is a trend of separation between the LG and CG groups (Fig. 2B, Supplementary Fig. S1).

### Gut microbiota composition and differential classification

**Fig. 3:**
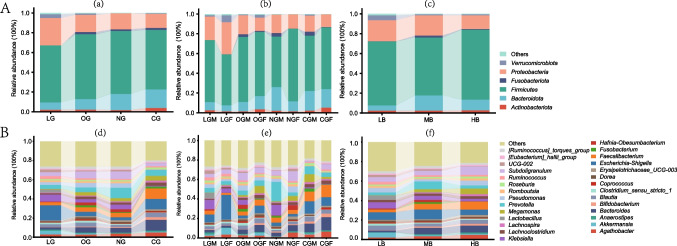
Species composition histograms for intergroup comparisons display phylum (**A**) and genus (**B**) with a mean relative abundance > 1%. (a–c) Represent the average relative abundance at the phylum level between groups in the three different grouping patterns, and (d–f) represent the average relative abundance at the genus level between groups in the three different grouping patterns, respectively

The composition of GM in subjects, based on different grouping patterns, is shown in Fig. 3. The data displays the microbial populations at the phylum level, with average species relative abundance above 1%. In all groups, the GM is dominated by *Firmicutes*, *Proteobacteria,* and *Bacteroidota*, which constitute the core of the phylogenetic tree at the phylum level, accounting for 90.26% (LGF) to 98.19% (NGF) (Fig. 3A). *Verrucomicrobiota* is relatively more abundant in the long-lived elderly population, particularly in long-lived females. The grouping based on BMI also confirmed the enrichment of *Verrucomicrobiota* in the elderly population with low BMI values. On the other hand, the LG shows a significant reduction in the relative abundance of *Fusobacteria*. At the genus level, 28 genera with average relative abundance above 1% are displayed (Fig. 3B). Among them, *Escherichia-Shigella*, *Prevotella, Bacteroides, Faecalibacterium,* and *Subdoligranulum* have an average relative abundance above 5% and are the dominant genera. The LG has the highest abundance of *Escherichia-Shigella* (15.94%), while the relative abundance in the OG and NG is significantly reduced to 4.53% and 2.94%, respectively. The *Subdoligranulum* is more abundant in the OG representing 7.11%, whereas the NG is enriched in *Prevotella* making up 10.69%. In addition, the CG shows an enrichment of *Bacteroides* comprising 11.19%. The grouping based on gender suggests that this genus appears to be more readily enriched in LGF. The gender-based grouping pattern has refined the inter-group differences. For instance, there is an increase in *Escherichia-Shigella* and *Akkermansia* in LGF compared to LGM, while *Lactobacillus* decreased.

**Fig. 4:**
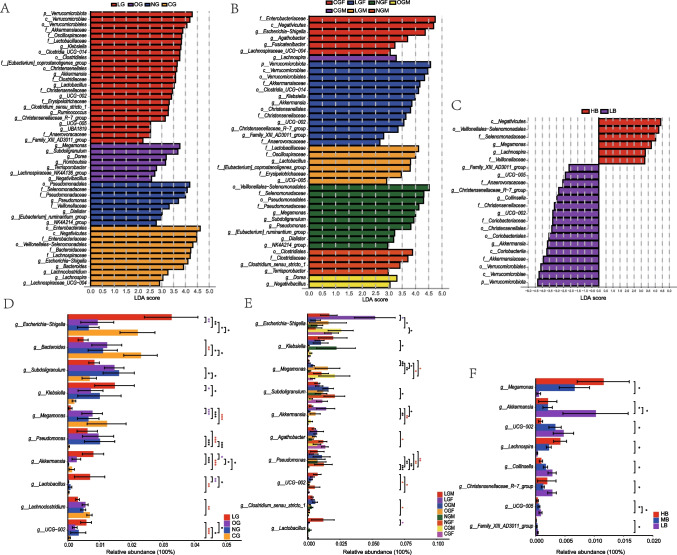
LEfSe analysis results of gut microbiota. (**A**–**C**) Histogram of LDA scores calculated for microbial community features showing inter-group differences in three different grouping patterns. The bar chart depicting the distribution of LDA values shows species with significant differences in abundance among different groups. The colors of the bars represent different groups, while the length of the bars indicates the magnitude of the impact of the various species. (**D**–**F**) Bar chart illustrating inter-group differences of the top 10 genera ranked by LDA scores in three grouping patterns. (**E**) only displays comparison results of the same gender. (**F**) only 8 different genera based on BMI grouping pattern. Red asterisks indicate significant differences between LG and CG, purple asterisks indicate significant differences between LG and OG. *, p < 0.05; **, p < 0.01; ***, p < 0.001

Based on the LEfSe results and the differential analysis of relative abundance at the genus level, it is evident that the LG shows significant enrichment in *Pseudomonas*, *Akkermansia*, *Lactobacillus*, and *Oscillospiraceae UCG-002* compared to the CG. Conversely, *Bacteroides*, *Megamonas*, and *Lachnoclostridium* are relatively enriched in the CG. When comparing the LG to the OG, *Escherichia-Shigella*, *Klebsiella*, *Akkermansia*, and *Lactobacillus* are significantly enriched in the LG, while *Megamonas* is relatively enriched in the OG. Among the genera that exhibited significant differences between LG and CG, *Akkermansia, Lactobacillus*, and *Megamonas* also displayed significant variances between LG and OG. *Akkermansia* and *Lactobacillus* were notably enriched in LG, whereas *Megamonas* experienced a significant decrease in LG. Additionally, we found that the relative abundance of Bacteroides was significantly reduced in the three groups in Changshou Town compared to the control group, while *Pseudomonas* exhibited the opposite trend. In terms of BMI grouping, the HB group is notably enriched in *Megamonas* and *Lachnospira* compared to the LB group. Conversely, *Akkermansia, Collinsella*, *Christensenellaceae_R-7_group, UCG-005*, *Family_XIII_AD3011_group*, and *Oscillospiraceae UCG-002* are relatively enriched in the LB group. Gender grouping analysis reveals consistent differences with previous findings, where trends in *Megamonas*, *Pseudomonas*, and *Oscillospiraceae UCG-002* between LGF-CGF and LGM-CGM are observed across both males and females. *Akkermansia, Agathobacter*, *Clostridium_sensu_stricto_1*, *Escherichia-Shigella*, and *Lactobacillus* exhibit significant differences in only one gender. However, it is important to note that this seemingly sex-related microbial distribution pattern did not show significant differences among the four pairs of subgroups. (FDR-adjusted *p* < 0.05) (Supplementary Table S2, Fig. 4).

### Functional pathway prediction of gut microbiota

**Fig. 5:**
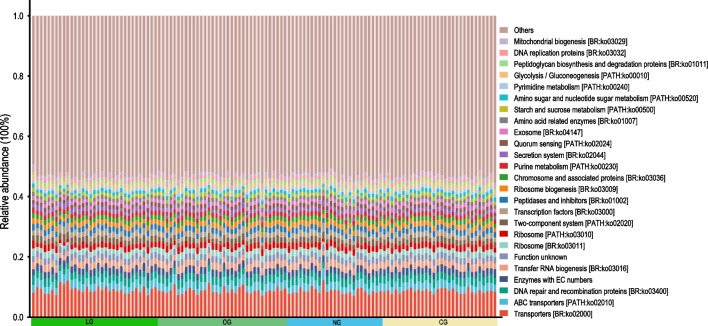
The bar chart displays the 25 KEGG Level 3 functional pathways with an average relative abundance predicted by PICRUSt2 in all samples exceeding 1%

PICRUSt2 was used to predict the potential function of the GM by comparing 16S rDNA sequences from fecal samples with known functional gene sequences from the KEGG database. A total of 52 secondary metabolic pathways (397 tertiary metabolic pathways) were obtained for all samples, with 150 pathways having an average abundance > 0.1% and 25 pathways having an average abundance > 1% (Fig. 5). Among them, 14 pathways were related to protein family’s metabolism, and 3 pathways were related to carbohydrate metabolism. Significant differences were found in certain tertiary metabolic pathways among various age groups (Supplementary Fig. S2). In the metabolic pathways with an average relative abundance > 0.1%, the abundance of the pentose phosphate pathway (PPP) and C5-branched dibasic acid metabolism in the GM of the LG was significantly lower than that in the OG and CG. The metabolic pathways related to longevity, such as the longevity regulating pathway-worm, pyruvate metabolism, and benzoate degradation in xenobiotics biodegradation, are significantly higher in the LG compared to the CG. On the other hand, the metabolic pathways related to galactose metabolism, *O*-antigen nucleotide sugar biosynthesis, and other glycan degradation are significantly lower in the CG. In the metabolic pathways with an average relative abundance greater than 0.01% but less than 0.1%, the abundance of the digestive system-related pancreatic secretion and salivary secretion metabolic pathways is significantly enriched in the LG. On the other hand, there is a significant difference in the relative abundance of the bile secretion pathway among the four groups, particularly in the CG, where it is significantly lower than in the other three groups. Additionally, we found that the enrichment level of the toluene degradation-related metabolic pathway is significantly higher in the LG compared to the other three groups. Interestingly, we also found that, compared to the HB group, the LB group shows a significant enrichment in the predicted metabolic pathways related to neurodegenerative diseases such as amyotrophic lateral sclerosis, Huntington’s disease, and pathways of neurodegeneration — multiple diseases.

### Redundancy analysis

**Fig. 6:**
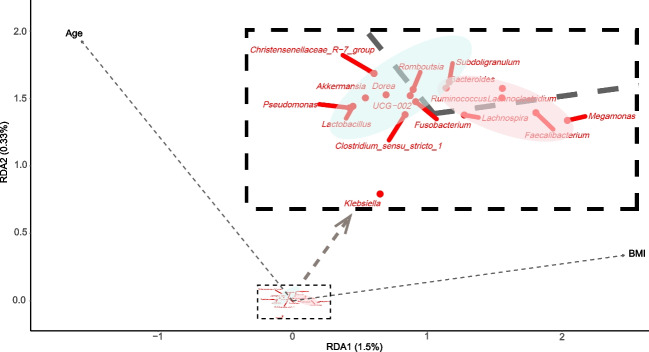
Redundancy analysis of the correlation between age, BMI value, and certain bacterial genera

As shown in Fig. 6, the redundancy analysis (RDA) results indicate that the genera positively correlated with age include Akkermansia, Christensenellaceae R-7 group, Romboutsia, Dorea, Pseudomonas, Lactobacillus, Fusobacterium, Clostridium sensu stricto 1, UCG-002. The genera positively correlated with BMI include Bacteroides, Faecalibacterium, Megamonas, Lachnoclostridium, and Lachnospira. Additionally, Subdoligranulum and Ruminococcus are positively correlated with both age and BMI.

## Discussion

Consistent with the research findings on GM in other long-lived elderly individuals, our results also indicate that long-lived elderly people have relatively high α diversity (Biagi et al. 2016; Kong et al. 2016, 2019; Ren et al. 2021). The rich diversity of GM may help maintain and restore stability (Lozupone et al. 2012). Conversely, a decrease in diversity due to aging and disease can make the microbiota less resilient to external threats, leading to chronic health issues (DeJong et al. 2020; Leite et al. 2021). This theory suggests a potential connection between increased gut microbiota diversity and longevity in long-lived elderly individuals. The results of the diversity analysis based on gender grouping patterns support the conclusions of Biagi et al. (2010), indicating that gender plays a minimal to negligible role in influencing aging-related gut microbiota. It is worth noting that the LB group exhibits significantly higher α-diversity compared to the other two groups. This group mainly comprises long-lived elderly individuals, suggesting that a lower BMI may be associated with health and longevity. This is consistent with Bhaskaran et al.’s (2018) theory of a J-shaped association between BMI and overall mortality. It is crucial to maintain a rich diversity in the gut microbiota as it is closely related to the stability and resilience of the gut microbiota ecosystem. In the elderly population, increased diversity may be beneficial for individuals to maintain healthy aging as a characteristic (Lozupone et al. 2012).

The microbial composition results showed that the dominant phyla in the gut microbiota are *Firmicutes*, *Proteobacteria*, and *Bacteroidetes,* which is consistent with previous reports (Kim et al. 2019; Tuikhar et al. 2019; Ren et al. 2021; Wu et al. 2022). Among them, *Firmicutes* and *Bacteroidetes* are the main components of the human gut microbiota, while *Proteobacteria* have been reported to increase in the elderly population over 70 years old but decrease in long-lived elderly individuals (Qin et al. 2010; Odamaki et al. 2016; Pang et al. 2023). Analysis of differential genera showed that *Akkermansia*, *Lactobacillus*, and *Megamonas* may be important gut microbiota markers distinguishing long-lived elderly individuals from younger elderly individuals. Similar to our results, several other studies on long-lived individuals have also found a significant enrichment of *Akkermansia* (Biagi et al. 2016; Kong et al. 2016; Tuikhar et al. 2019). *Akkermansia* is a well-studied genus in longevity research and is associated with potential negative correlations with various diseases such as obesity, diabetes, and cardiovascular diseases (Pellegrino et al. 2023). Recent fecal microbiota transplantation experiments by Bárcena et al. (2019) have confirmed the important role of *Akkermansia muciniphila* in improving the health and lifespan of prematurely aging mice. These findings suggest that individuals with a long lifespan may benefit from a high abundance of *Akkermansia* in their gut microbiota. The ability of *A. muciniphila* to degrade mucin not only helps maintain gut barrier function but also plays a crucial role in disease prevention mechanisms, such as inhibiting inflammation, regulating the immune system, and improving metabolism through the production of short-chain fatty acids (SCFAs) (Hasani et al. 2021; Lakshmanan et al. 2022; Wosińska et al. 2023). This also explains the significant enrichment of *Akkermansia* in elderly individuals with low BMI values. Additionally, no significant differences in *Akkermansia* were found between males and females, which is consistent with a study by Guo et al. (2016) in a population from southern China. This suggests that *Akkermansia* may be influenced by factors such as age and geographical environment rather than gender, or it may only have a weak association with gender (Collado et al. 2007). *Lactobacillus* is a common probiotic in the human gut, but its quantity gradually decreases with age (Zhang et al. 2018; Samtiya et al. 2022). In this study, there was a significant enrichment in the long-lived elderly group, similar to previous research findings (Kim et al. 2019; Wu et al. 2022). In fact, the potential association between *Lactobacillus* and longevity and health was proposed over a century ago (Mackowiak 2013). Recent studies have not only reported the important roles of *Lactobacillus* in gut homeostasis, immune regulation, inflammation, oxidative stress, and nervous system regulation, but also confirmed its significant potential in extending lifespan (Grompone et al. 2012; Kumaree et al. 2023; Zhao et al. 2023). However, the specific mechanisms of *Lactobacillus* in delaying aging and maintaining healthy aging are still unclear. Interestingly, the increase in *Lactobacillus* seems to be associated with *Akkermansia*. The increase in *Akkermansia* not only promotes *Lactobacillus* but also suppresses *Bacteroides* (Wang et al. 2020). This explanation clarifies the enrichment of *Akkermansia* and *Lactobacillus* in long-lived elderly individuals, while *Bacteroides* significantly decreases. However, this contradicts previous reports of *Bacteroides* enrichment in long-lived elderly individuals. (Park et al. 2015b; Li et al. 2023; Pang et al. 2023). *Bacteroides* are crucial microorganisms in the human intestine. Despite the potential to be pathogenic, this bacterium often plays a beneficial role. It not only helps in the digestion and absorption of nutrients, but the deficiency of *Bacteroides* is closely related to many diseases (Wexler 2007; Wang et al. 2021; Zafar and Saier 2021). This evidence of the health benefits of *Bacteroides* seems to support the possibility that they are enriched in the intestines of long-lived individuals. However, it should be noted that factors such as geographical location and dietary differences are also important considerations influencing its distribution (De Filippis et al. 2016; Gorvitovskaia et al. 2016; Mobeen et al. 2018). In addition to *Bacteroides*, *Megamonas* were also significantly reduced in the gut microbiota of long-lived seniors. *Megamonas* has been reported as a core genus in the intestines of Asian populations (Yachida et al. 2019). It is not only more prevalent in males but also decreases with age and frailty (Takagi et al. 2019; Xu et al. 2021; Wu et al. 2022; Yan et al. 2022). The significant increase in *Megamonas* in patients with obesity and fatty liver disease seems to support its significant enrichment in the HB group, whereas it was significantly reduced in the high animal fat dietary pattern (Wan et al. 2019). These results indicate the potential role of *Megamonas* in metabolism. However, conflicting findings in different disease-related reports render the role of *Megamonas* in human health inconclusive (Yang et al. 2023).

The functional prediction results based on PICRUSt2 did not find any significant differences in metabolic pathways directly related to longevity between the long-lived elderly group and the offspring group. The significant differences were concentrated in xenobiotic biodegradation and metabolism, digestive system, and carbohydrate metabolism. Compared to the younger elderly group, the long-lived elderly group exhibited a greater enrichment of metabolic pathways related to xenobiotics biodegradation in the gut. This was primarily observed in the significant enrichment of toluene-related degradation pathways, as well as higher levels of benzoate, nitrotoluene, and aminobenzoate degradation related pathways compared to the other groups. This is consistent with the current theory that regional restrictions on the activity trajectory of the long-lived elderly population, along with their longer history of exogenous exposure, gradually accumulate in the body with age (Rampelli et al. 2020). It is well known that aging leads to a gradual decline in organism function, including changes in the metabolic capacity and secretions of the salivary glands, pancreas, and gallbladder in the digestive system (Khalil et al. 1985; Krøll 2012; Toan and Ahn 2021). These changes, in turn, affect the composition of the GM. We found that the metabolic pathways related to pancreatic and salivary secretion were more enriched in the long-lived elderly group compared to the offspring group and the control group. This seemingly contradictory result may be one of the characteristics of the long-lived elderly population who maintain healthy longevity. Certain antioxidants, proteins, and immune factors in secretions may have protective and health-promoting effects. However, the LG appeared to be unable to reverse the decline in bile acid synthesis and bile flow caused by aging, and their associated metabolic pathways were significantly lower than those of CG and OG. Interestingly, we found that the metabolic pathways related to bile secretion were significantly enriched in all three groups in Changshou Town compared to the CG. This may be associated with geographical location or genetic factors, but the specific mechanism is still unclear, and further research is needed. Compared to CG and OG, the pentose phosphate pathway (PPP) and C5-branched dibasic acid metabolism in carbohydrate metabolism pathway are significantly reduced in LG. The nicotinamide adenine dinucleotide phosphate (NADPH) generated in the oxidative phase of the PPP can enhance oxidative stress tolerance and extend the host's lifespan (Bradshaw 2019; Shen et al. 2023). Moreover, the PPP, which is a crucial pathway in central carbon metabolism, can be associated with the extension of lifespan by influencing mitochondrial function (Bennett et al. 2017). The C5-branched dibasic acid metabolism pathway is positively correlated with the production of SCFA (Wu et al. 2021b; Fang et al. 2022). SCFA plays an important role in maintaining intestinal barrier function and host health. Pyruvic acid, as the main precursor of the three major short-chain fatty acids (acetate, propionate, and butyrate), plays a key role in glycolysis and the carbon cycle (Koh et al. 2016). Interestingly, both the LG and OG showed significantly enriched pyruvate metabolism compared to the CG, which is consistent with the findings of Wu et al. (2019) in centenarians in Sardinia. This indicates that the GM of the long-lived elderly population may have a higher ability to produce SCFA to maintain health. In addition, we found that the ability of galactose metabolism in the three elderly groups in Changshou Town was significantly lower than that of the control group. Although this study bears similarities to the previous study conducted by Wu et al. (2019), the findings in this research may be attributed more to geographical location and dietary variations. Therefore, further research focusing on diet interventions may be necessary in the future to substantiate these results. In addition, no significant differences were observed in neurodegenerative diseases closely related to aging among age groups, but the analysis of grouping results based on BMI showed that samples with low BMI values were more enriched in the metabolic pathways of amyotrophic lateral sclerosis, Huntington disease, and pathways of neurodegeneration — multiple diseases, indicating a higher possibility of developing neurodegenerative diseases. Although the “lean type” elderly population mentioned earlier has unique advantages in maintaining health, this result suggests to some extent that they may not be able to reverse neurodegenerative diseases caused by aging. It is important to note that there are more individuals with longer lifespans in the LB group, which needs to be taken into consideration. Although PICRUSt has been widely used in microbial research, it is important to clarify that it can only predict the functions of known microbial genes. It is also worth noting that horizontal gene transfer is common among bacteria, which means that its predictive function may not accurately reflect the actual situation (Sun et al. 2020).

In conclusion, our research shows that long-lived populations and elderly populations with low BMI have unique characteristics in their GM. However, longevity is not only related to GM but is also influenced by genetic and environmental factors. Additionally, GM is closely associated with dietary behavior. Although we attempted to exclude influences such as disease from the study population, it is unrealistic to completely eliminate all confounding variables in practice. Additionally, there is currently no clear definition of age groups in longevity research. Therefore, standardization is necessary in the future to facilitate comparative studies between long-lived populations in different regions and to further investigate specific microbial populations associated with longevity. Furthermore, functional predictions of GM based on PICRUSt can only partially reflect the true information of the samples and cannot fully substitute for metagenomic research. Therefore, in the future, it will be necessary to further utilize multi-omics technologies, such as metagenomics and metabolomics, to comprehensively investigate the mechanisms underlying the interaction between GM and healthy aging.

## Supplementary Information

Below is the link to the electronic supplementary material.

Supplementary file1 (PDF 1480 KB)

## Data Availability

The raw read sequences of the 16S rRNA genes in this study are publicly available in the NCBI SRA depository within BioProject PRJNA1021027 and PRJNA985413, and the BioSample accession numbers can be found in the supplementary material.
