# Supplementary material for: Structural characteristics of gut microbiota in longevity from Changshou town, Hubei, China
Source: Appl Microbiol Biotechnol. 2024 Apr 15;108:300. doi: 10.1007/s00253-024-13140-3 (PMC11018559; doi:10.1007/s00253-024-13140-3)
Supplement: Supplementary file 1 [file 253_2024_13140_MOESM1_ESM.pdf]

Journal: Applied Microbiology and Biotechnology

Supplementary Material

Structural Characteristics of Gut Microbiota in Longevity Elderly from Changshou Town, Hubei, China

Xu Ai [1,†], Yu Liu [1,†], Jinrong Shi [1], Xiongwei Xie [1], Linzi Li [1], Rui Duan [1], Yongling Lv [2], Kai Xiong [2], Yuanxin Miao [3,*], Yonglian Zhang [1,*]

[1] Jingmen Central Hospital, Hubei Clinical Medical Research Center for Functional Colorectal Diseases, Jingmen 448000, Hubei, China

[2] Maintainbiotech. Ltd. (Wuhan)., Wuhan 430000, Hubei, China

[3] Research Institute of Agricultural Biotechnology, Jingchu University of Technology, Jingmen 448000, Hubei, China

† These authors contributed equally to this work and should be considered co-first authors.

[*] Correspondence to: Yonglian Zhang, email: zhdzyzyl1983@163.com; Yuanxin Miao, email: Yxmiao@jcut.edu.cn

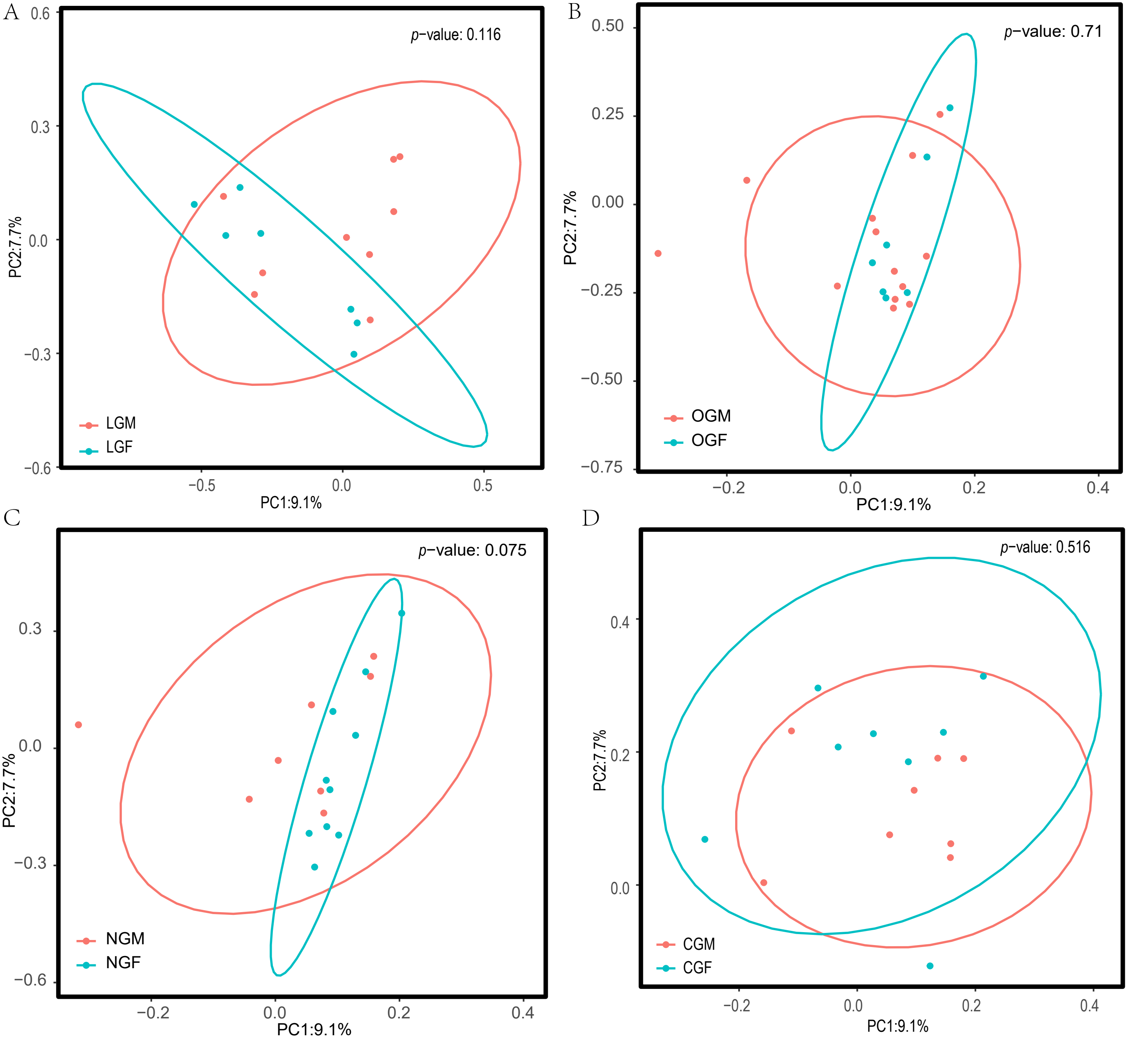

Fig. S1. Beta diversity between gender groups. (A) LGM, longevity group male; LGF, longevity group female; (B) OGM, offspring group male; OGF, offspring group female; (C) NGM, neighbor group male; NGF, neighbor group female; (D) CGM, control group male; CGF, control group female.

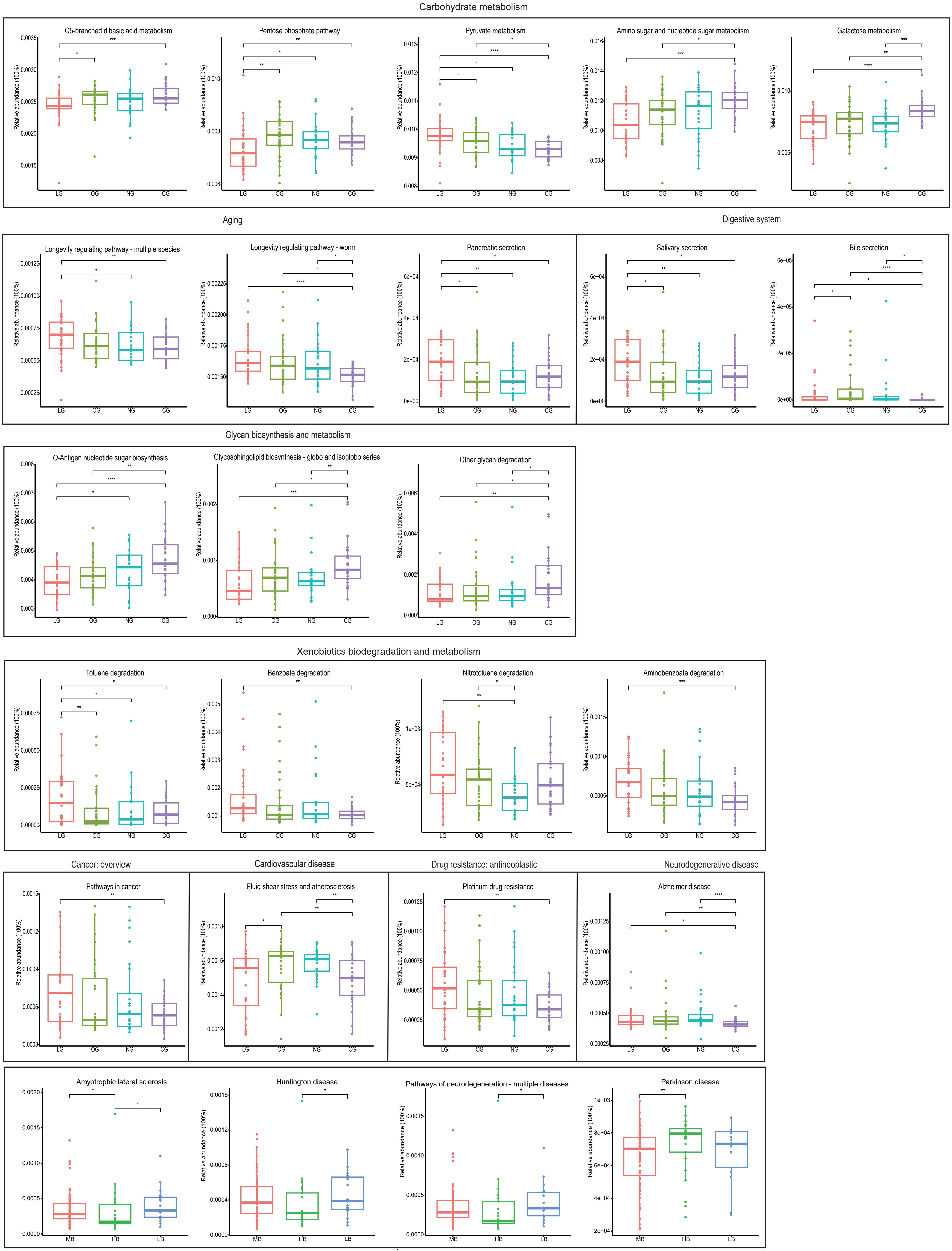

Figure S2: Relative abundance of functional prediction pathways among different groups.

Table S1. Sample sequencing data information.

Group	Sample ID	Accession number	Raw reads	High quality reads	R	Sum length	Average	Minimum	Maximum
LG	S0187693981	SAMN35796715	129,499	94,224	R1	29,399,999	227	45	231
					R2	29,131,588	225	1	228
	S0187694131	SAMN35796717	62,762	44,647	R1	14,249,659	227	45	231
					R2	14,113,881	224.9	21	228
	S0187694280	SAMN35796718	80,867	52,734	R1	18,353,912	227	67	230
					R2	18,186,309	224.9	34	229
	S0187697977	SAMN35796719	79,865	46,358	R1	18,125,961	227	22	230
					R2	17,963,582	224.9	22	229
	S0187698312	SAMN35796726	75,999	33,572	R1	17,241,873	226.9	48	231
					R2	17,085,043	224.8	1	228
	S0187698419	SAMN35796728	75,674	59,094	R1	17,175,319	227	83	231
					R2	17,015,419	224.9	34	229
	S0187698483	SAMN35796731	109,550	61,305	R1	24,860,453	226.9	67	233
					R2	24,637,272	224.9	26	228
	S0187702127	SAMN35796735	85,968	59,364	R1	19,511,327	227	67	232
					R2	19,332,098	224.9	56	228
	S0187702317	SAMN35796739	50,034	34,609	R1	11,356,580	227	104	231
					R2	11,251,578	224.9	57	229
	S0187702334	SAMN35796740	50,238	39,703	R1	11,401,891	227	130	231
					R2	11,296,055	224.9	130	228
	S0187702511	SAMN35796745	61,033	39,326	R1	13,852,637	227	67	232
					R2	13,726,538	224.9	130	228
	S0187705250	SAMN35796748	115,888	69,764	R1	26,282,877	226.8	67	231
					R2	26,054,018	224.8	40	232
	S0187705314	SAMN35796749	58,118	42,022	R1	13,188,615	226.9	62	229
					R2	13,068,545	224.9	62	228
	S0187705376	SAMN35796750	105,699	59,512	R1	23,989,432	227	6	234
					R2	23,770,754	224.9	6	228
	S0187713718	SAMN35796756	94,193	57,206	R1	21,346,268	226.6	67	231
					R2	21,183,923	224.9	34	228
	S0187714258	SAMN35796760	55,974	44,489	R1	12,704,484	227	125	231
					R2	12,589,821	224.9	122	228
	S0187714569	SAMN35796762	94,484	62,442	R1	21,448,846	227	67	231
					R2	21,246,977	224.9	21	230

	S0187714872	SAMN35796770	59,002	44,324	R1	13,392,274	227	67	234
					R2	13,269,599	224.9	150	228
	S019934620	SAMN35796783	105,712	60,554	R1	23,991,318	226.9	67	233
					R2	23,769,633	224.9	148	228
	S019934623	SAMN35796784	98,127	53,528	R1	22,271,006	227	67	234
					R2	22,069,147	224.9	56	232
	S019934636	SAMN35796785	91,207	50,992	R1	20,685,522	226.8	49	234
					R2	20,498,613	224.7	5	230
	S019934710	SAMN35796788	91,603	34,744	R1	20,785,759	226.9	67	232
					R2	20,602,032	224.9	122	233
	S019934759	SAMN35796790	72,485	42,043	R1	16,451,272	227	49	233
					R2	16,303,300	224.9	19	228
	S019934859	SAMN35796793	70,499	57,749	R1	16,000,598	227	21	233
					R2	15,852,299	224.9	21	228
	S019934988	SAMN35796797	77,706	30,112	R1	17,636,107	227	45	231
					R2	17,479,671	224.9	45	228
	S019935040	SAMN35796798	91,675	46,972	R1	20,804,173	226.9	47	234
					R2	20,620,945	224.9	49	229
	S019935054	SAMN35796800	117,151	71,904	R1	26,594,143	227	71	231
					R2	26,342,379	224.9	26	229
	S019935103	SAMN35796803	88,431	56,650	R1	20,068,605	226.9	13	232
					R2	19,888,967	224.9	1	231
	S019935142	SAMN35796806	127,467	62,993	R1	28,927,840	226.9	33	230
					R2	28,664,982	224.9	33	228
	S019935182	SAMN35796807	58,994	46,775	R1	13,382,610	226.8	122	233
					R2	13,267,492	224.9	122	229
	S019935183	SAMN35796808	72,373	51,052	R1	16,413,883	226.8	67	232
					R2	16,275,135	224.9	70	229
	S019935212	SAMN35796810	116,849	66,515	R1	26,514,376	226.9	67	230
					R2	26,277,865	224.9	56	229
	S019935228	SAMN35796811	95,694	56,498	R1	21,700,836	226.8	45	233
					R2	21,519,800	224.9	34	231

Table S2. Genera with significant differences between different groups.

Comparison	Taxa	Group	*p*.value	*p*.BH
LG - CG	*k__Bacteria|p__Bacteroidota|c__Bacteroidia|o__Bacteroidales|f__Bacteroidaceae|g__Bacteroides*	CG	0.000161261	0.002217797
LG - CG	*k__Bacteria|p__Firmicutes|c__Clostridia|o__Oscillospirales|f__Ruminococcaceae|g__Faecalibacterium*	CG	0.002253059	0.014747
LG - CG	*k__Bacteria|p__Firmicutes|c__Negativicutes|o__Veillonellales-Selenomonadales|f__Selenomonadaceae|g__Megamonas*	CG	3.82E-07	1.58E-05
LG - CG	*k__Bacteria|p__Proteobacteria|c__Gammaproteobacteria|o__Pseudomonadales|f__Pseudomonadaceae|g__Pseudomonas*	LG	1.64E-08	1.71E-06
LG - CG	*k__Bacteria|p__Firmicutes|c__Clostridia|o__Lachnospirales|f__Lachnospiraceae|g__Lachnoclostridium*	CG	5.82E-05	0.001011315
LG - CG	*k__Bacteria|p__Firmicutes|c__Clostridia|o__Oscillospirales|f__Ruminococcaceae|g__Ruminococcus*	LG	0.001188023	0.010819943
LG - CG	*k__Bacteria|p__Verrucomicrobiota|c__Verrucomicrobiae|o__Verrucomicrobiales|f__Akkermansiaceae|g__Akkermansia*	LG	1.80E-06	4.56E-05
LG - CG	*k__Bacteria|p__Firmicutes|c__Clostridia|o__Peptostreptococcales-Tissierellales|f__Peptostreptococcaceae|g__Romboutsia*	LG	0.003935484	0.021290321
LG - CG	*k__Bacteria|p__Firmicutes|c__Clostridia|o__Oscillospirales|f__Oscillospiraceae|g__UCG-002*	LG	5.89E-07	2.16E-05
LG - CG	*k__Bacteria|p__Firmicutes|c__Clostridia|o__Clostridiales|f__Clostridiaceae|g__Clostridium_sensu_stricto_1*	LG	7.52E-05	0.00124056
LG - CG	*k__Bacteria|p__Firmicutes|c__Clostridia|o__Lachnospirales|f__Lachnospiraceae|g__Lachnospira*	CG	0.008927433	0.037373964
LG - CG	*k__Bacteria|p__Firmicutes|c__Clostridia|o__Lachnospirales|f__Lachnospiraceae|g__Anaerostipes*	CG	0.006117569	0.029688204
LG - CG	*k__Bacteria|p__Firmicutes|c__Bacilli|o__Lactobacillales|f__Lactobacillaceae|g__Lactobacillus*	LG	0.000300927	0.003677999
LG - CG	*k__Bacteria|p__Firmicutes|c__Clostridia|o__Christensenellales|f__Christensenellaceae|g__Christensenellaceae_R-7_group*	LG	1.73E-07	9.53E-06
LG - CG	*k__Bacteria|p__Firmicutes|c__Negativicutes|o__Veillonellales-Selenomonadales|f__Veillonellaceae|g__Dialister*	CG	0.005100146	0.026297628
LG - CG	*k__Bacteria|p__Firmicutes|c__Clostridia|o__Lachnospirales|f__Lachnospiraceae|g__Lachnospiraceae_UCG-004*	CG	2.53E-05	0.000521779
LG - CG	*k__Bacteria|p__Firmicutes|c__Clostridia|o__Oscillospirales|f__Oscillospiraceae|g__NK4A214_group*	LG	1.10E-06	3.62E-05
LG - CG	*k__Bacteria|p__Firmicutes|c__Clostridia|o__Lachnospirales|f__Lachnospiraceae|g__[Eubacterium]_ruminantium_group*	LG	0.000161294	0.002217797
LG - CG	*k__Bacteria|p__Firmicutes|c__Clostridia|o__Oscillospirales|f__Ruminococcaceae|g__Negativibacillus*	LG	0.002356404	0.014954102
LG - CG	*k__Bacteria|p__Firmicutes|c__Clostridia|o__Peptostreptococcales-Tissierellales|f__Peptostreptococcaceae|g__Terrisporobacter*	LG	0.001525921	0.012487125
LG - CG	*k__Bacteria|p__Firmicutes|c__Clostridia|o__Oscillospirales|f__Oscillospiraceae|g__UCG-005*	LG	1.95E-05	0.000428304
LG - CG	*k__Bacteria|p__Firmicutes|c__Clostridia|o__Oscillospirales|f__Ruminococcaceae|g__UBA1819*	LG	0.001093112	0.010565873
LG - CG	*k__Bacteria|p__Firmicutes|c__Clostridia|o__Peptostreptococcales-Tissierellales|f__Anaerovoracaceae|g__Family_XIII_AD3011_group*	LG	1.33E-06	3.99E-05
LG - NG	*k__Bacteria|p__Proteobacteria|c__Gammaproteobacteria|o__Enterobacterales|f__Enterobacteriaceae|g__Escherichia-Shigella*	LG	0.000241801	0.003191769
LG - NG	*k__Bacteria|p__Proteobacteria|c__Gammaproteobacteria|o__Enterobacterales|f__Enterobacteriaceae|g__Klebsiella*	LG	0.011322016	0.046703316
LG - NG	*k__Bacteria|p__Firmicutes|c__Negativicutes|o__Veillonellales-Selenomonadales|f__Selenomonadaceae|g__Megamonas*	NG	2.35E-07	1.11E-05
LG - NG	*k__Bacteria|p__Verrucomicrobiota|c__Verrucomicrobiae|o__Verrucomicrobiales|f__Akkermansiaceae|g__Akkermansia*	LG	1.45E-06	3.99E-05
LG - NG	*k__Bacteria|p__Firmicutes|c__Clostridia|o__Oscillospirales|f__Oscillospiraceae|g__UCG-002*	LG	0.004449522	0.023682938
LG - NG	*k__Bacteria|p__Firmicutes|c__Clostridia|o__Lachnospirales|f__Lachnospiraceae|g__Lachnospira*	NG	0.002159048	0.014747
LG - NG	*k__Bacteria|p__Firmicutes|c__Bacilli|o__Lactobacillales|f__Lactobacillaceae|g__Lactobacillus*	LG	0.008512224	0.037150687
LG - NG	*k__Bacteria|p__Firmicutes|c__Clostridia|o__Christensenellales|f__Christensenellaceae|g__Christensenellaceae_R-7_group*	LG	0.001664621	0.012870531
LG - NG	*k__Bacteria|p__Firmicutes|c__Negativicutes|o__Veillonellales-Selenomonadales|f__Veillonellaceae|g__Dialister*	NG	0.003337888	0.018991432

LG - NG	*k__Bacteria|p__Firmicutes|c__Clostridia|o__Lachnospirales|f__Lachnospiraceae|g__Lachnospiraceae_UCG-004*	NG	0.003076256	0.017809906
LG - NG	*k__Bacteria|p__Firmicutes|c__Clostridia|o__Lachnospirales|f__Lachnospiraceae|g__[Eubacterium]_ruminantium_group*	NG	0.002265889	0.014747
LG - NG	*k__Bacteria|p__Firmicutes|c__Clostridia|o__Oscillospirales|f__Oscillospiraceae|g__UCG-005*	LG	0.00107985	0.010565873
LG - NG	*k__Bacteria|p__Firmicutes|c__Clostridia|o__Oscillospirales|f__Ruminococcaceae|g__UBA1819*	LG	0.003487373	0.019505648
LG - NG	*k__Bacteria|p__Firmicutes|c__Clostridia|o__Peptostreptococcales-Tissierellales|f__Anaerovoracaceae|g__Family_XIII_AD3011_group*	LG	0.001245933	0.010819943
LG - OG	*k__Bacteria|p__Proteobacteria|c__Gammaproteobacteria|o__Enterobacterales|f__Enterobacteriaceae|g__Escherichia-Shigella*	LG	0.000148385	0.002217797
LG - OG	*k__Bacteria|p__Bacteroidota|c__Bacteroidia|o__Bacteroidales|f__Prevotellaceae|g__Prevotella*	OG	0.008845192	0.037373964
LG - OG	*k__Bacteria|p__Proteobacteria|c__Gammaproteobacteria|o__Enterobacterales|f__Enterobacteriaceae|g__Klebsiella*	LG	0.001120623	0.010565873
LG - OG	*k__Bacteria|p__Firmicutes|c__Negativicutes|o__Veillonellales-Selenomonadales|f__Selenomonadaceae|g__Megamonas*	OG	1.55E-09	2.56E-07
LG - OG	*k__Bacteria|p__Verrucomicrobiota|c__Verrucomicrobiae|o__Verrucomicrobiales|f__Akkermansiaceae|g__Akkermansia*	LG	0.002795118	0.016934436
LG - OG	*k__Bacteria|p__Firmicutes|c__Clostridia|o__Lachnospirales|f__Lachnospiraceae|g__Dorea*	OG	0.005835542	0.028742224
LG - OG	*k__Bacteria|p__Firmicutes|c__Clostridia|o__Lachnospirales|f__Lachnospiraceae|g__Lachnospira*	OG	0.008215252	0.03713744
LG - OG	*k__Bacteria|p__Firmicutes|c__Bacilli|o__Lactobacillales|f__Lactobacillaceae|g__Lactobacillus*	LG	0.000657888	0.007036036
LG - OG	*k__Bacteria|p__Firmicutes|c__Clostridia|o__Lachnospirales|f__Lachnospiraceae|g__Lachnospiraceae_NK4A136_group*	OG	0.000660961	0.007036036
LG - OG	*k__Bacteria|p__Firmicutes|c__Clostridia|o__Christensenellales|f__Christensenellaceae|g__Christensenellaceae_R-7_group*	LG	0.005189553	0.026346963
LG - OG	*k__Bacteria|p__Firmicutes|c__Clostridia|o__Lachnospirales|f__Lachnospiraceae|g__Lachnospiraceae_UCG-004*	OG	0.001551431	0.012487125
LG - OG	*k__Bacteria|p__Firmicutes|c__Clostridia|o__Oscillospirales|f__Ruminococcaceae|g__UBA1819*	LG	0.007708198	0.035826834
NG - CG	*k__Bacteria|p__Proteobacteria|c__Gammaproteobacteria|o__Enterobacterales|f__Enterobacteriaceae|g__Escherichia-Shigella*	CG	0.00382219	0.021022042
NG - CG	*k__Bacteria|p__Bacteroidota|c__Bacteroidia|o__Bacteroidales|f__Bacteroidaceae|g__Bacteroides*	CG	0.006667829	0.031889619
NG - CG	*k__Bacteria|p__Firmicutes|c__Clostridia|o__Oscillospirales|f__Ruminococcaceae|g__Subdoligranulum*	NG	0.002606888	0.016231567
NG - CG	*k__Bacteria|p__Proteobacteria|c__Gammaproteobacteria|o__Pseudomonadales|f__Pseudomonadaceae|g__Pseudomonas*	NG	8.94E-08	5.90E-06
NG - CG	*k__Bacteria|p__Firmicutes|c__Clostridia|o__Peptostreptococcales-Tissierellales|f__Peptostreptococcaceae|g__Romboutsia*	NG	0.001241927	0.010819943
NG - CG	*k__Bacteria|p__Firmicutes|c__Clostridia|o__Lachnospirales|f__Lachnospiraceae|g__Dorea*	NG	0.002279082	0.014747
NG - CG	*k__Bacteria|p__Firmicutes|c__Clostridia|o__Christensenellales|f__Christensenellaceae|g__Christensenellaceae_R-7_group*	NG	0.007421455	0.034986859
NG - CG	*k__Bacteria|p__Firmicutes|c__Clostridia|o__Oscillospirales|f__Oscillospiraceae|g__NK4A214_group*	NG	0.000295582	0.003677999
NG - CG	*k__Bacteria|p__Firmicutes|c__Clostridia|o__Lachnospirales|f__Lachnospiraceae|g__[Eubacterium]_ventriosum_group*	NG	0.002822406	0.016934436
NG - CG	*k__Bacteria|p__Firmicutes|c__Clostridia|o__Lachnospirales|f__Lachnospiraceae|g__[Eubacterium]_ruminantium_group*	NG	2.08E-08	1.71E-06
NG - CG	*k__Bacteria|p__Firmicutes|c__Clostridia|o__Oscillospirales|f__Ruminococcaceae|g__Negativibacillus*	NG	0.001780607	0.013354555
NG - CG	*k__Bacteria|p__Firmicutes|c__Clostridia|o__Lachnospirales|f__Lachnospiraceae|g__Lachnospiraceae_UCG-001*	NG	0.008363692	0.037150687
OG - CG	*k__Bacteria|p__Proteobacteria|c__Gammaproteobacteria|o__Enterobacterales|f__Enterobacteriaceae|g__Escherichia-Shigella*	CG	0.002207455	0.014747
OG - CG	*k__Bacteria|p__Bacteroidota|c__Bacteroidia|o__Bacteroidales|f__Bacteroidaceae|g__Bacteroides*	CG	0.00225966	0.014747
OG - CG	*k__Bacteria|p__Firmicutes|c__Clostridia|o__Oscillospirales|f__Ruminococcaceae|g__Subdoligranulum*	OG	0.002106306	0.014747
OG - CG	*k__Bacteria|p__Proteobacteria|c__Gammaproteobacteria|o__Pseudomonadales|f__Pseudomonadaceae|g__Pseudomonas*	OG	3.41E-10	1.12E-07
OG - CG	*k__Bacteria|p__Verrucomicrobiota|c__Verrucomicrobiae|o__Verrucomicrobiales|f__Akkermansiaceae|g__Akkermansia*	OG	0.008068157	0.036979053

<table>
<tr><td>OG - CG</td><td>k__Bacteria|p__Firmicutes|c__Clostridia|o__Peptostreptococcales-Tissierellales|f__Peptostreptococcaceae|g__Romboutsia</td><td>OG</td><td>0.000631831</td><td>0.007036036</td></tr>
<tr><td>OG - CG</td><td>k__Bacteria|p__Firmicutes|c__Clostridia|o__Oscillospirales|f__Oscillospiraceae|g__UCG-002</td><td>OG</td><td>0.001355196</td><td>0.011467043</td></tr>
<tr><td>OG - CG</td><td>k__Bacteria|p__Firmicutes|c__Clostridia|o__Clostridiales|f__Clostridiaceae|g__Clostridium_sensu_stricto_1</td><td>OG</td><td>0.000855102</td><td>0.008818239</td></tr>
<tr><td>OG - CG</td><td>k__Bacteria|p__Firmicutes|c__Clostridia|o__Lachnospirales|f__Lachnospiraceae|g__Dorea</td><td>OG</td><td>1.72E-05</td><td>0.00040519</td></tr>
<tr><td>OG - CG</td><td>k__Bacteria|p__Firmicutes|c__Clostridia|o__Christensenellales|f__Christensenellaceae|g__Christensenellaceae_R-7_group</td><td>OG</td><td>0.001677069</td><td>0.012870531</td></tr>
<tr><td>OG - CG</td><td>k__Bacteria|p__Firmicutes|c__Negativicutes|o__Veillonellales-Selenomonadales|f__Veillonellaceae|g__Dialister</td><td>CG</td><td>0.0089471</td><td>0.037373964</td></tr>
<tr><td>OG - CG</td><td>k__Bacteria|p__Firmicutes|c__Clostridia|o__Oscillospirales|f__Oscillospiraceae|g__NK4A214_group</td><td>OG</td><td>0.000361361</td><td>0.004258894</td></tr>
<tr><td>OG - CG</td><td>k__Bacteria|p__Firmicutes|c__Clostridia|o__Lachnospirales|f__Lachnospiraceae|g__[Eubacterium]_ruminantium_group</td><td>OG</td><td>4.17E-05</td><td>0.000764626</td></tr>
<tr><td>OG - CG</td><td>k__Bacteria|p__Firmicutes|c__Clostridia|o__Oscillospirales|f__Ruminococcaceae|g__Negativibacillus</td><td>OG</td><td>0.000132957</td><td>0.00208932</td></tr>
<tr><td>OG - CG</td><td>k__Bacteria|p__Firmicutes|c__Clostridia|o__Peptostreptococcales-Tissierellales|f__Peptostreptococcaceae|g__Terrisporobacter</td><td>OG</td><td>3.34E-05</td><td>0.000647983</td></tr>
<tr><td>OG - CG</td><td>k__Bacteria|p__Firmicutes|c__Clostridia|o__Oscillospirales|f__Oscillospiraceae|g__UCG-005</td><td>OG</td><td>0.005448512</td><td>0.027242561</td></tr>
<tr><td>OG - CG</td><td>k__Bacteria|p__Firmicutes|c__Bacilli|o__Erysipelotrichales|f__Erysipelotrichaceae|g__Turicibacter</td><td>OG</td><td>0.005037256</td><td>0.026297628</td></tr>
<tr><td>OG - NG</td><td>k__Bacteria|p__Verrucomicrobiota|c__Verrucomicrobiae|o__Verrucomicrobiales|f__Akkermansiaceae|g__Akkermansia</td><td>OG</td><td>0.002918995</td><td>0.017201218</td></tr>
<tr><td>OG - NG</td><td>k__Bacteria|p__Firmicutes|c__Negativicutes|o__Veillonellales-Selenomonadales|f__Veillonellaceae|g__Dialister</td><td>NG</td><td>0.008555916</td><td>0.037150687</td></tr>
<tr><td>LGF - CGF</td><td>k__Bacteria|p__Firmicutes|c__Clostridia|o__Oscillospirales|f__Ruminococcaceae|g__Faecalibacterium</td><td>CGF</td><td>0.000577781</td><td>0.017795664</td></tr>
<tr><td>LGF - CGF</td><td>k__Bacteria|p__Firmicutes|c__Clostridia|o__Lachnospirales|f__Lachnospiraceae|g__Agathobacter</td><td>CGF</td><td>0.00196295</td><td>0.038265109</td></tr>
<tr><td>LGF - CGF</td><td>k__Bacteria|p__Firmicutes|c__Negativicutes|o__Veillonellales-Selenomonadales|f__Selenomonadaceae|g__Megamonas</td><td>CGF</td><td>0.000329614</td><td>0.01557348</td></tr>
<tr><td>LGF - CGF</td><td>k__Bacteria|p__Proteobacteria|c__Gammaproteobacteria|o__Pseudomonadales|f__Pseudomonadaceae|g__Pseudomonas</td><td>LGF</td><td>8.26E-05</td><td>0.007907282</td></tr>
<tr><td>LGF - CGF</td><td>k__Bacteria|p__Verrucomicrobiota|c__Verrucomicrobiae|o__Verrucomicrobiales|f__Akkermansiaceae|g__Akkermansia</td><td>LGF</td><td>0.000154405</td><td>0.009907657</td></tr>
<tr><td>LGF - CGF</td><td>k__Bacteria|p__Firmicutes|c__Clostridia|o__Oscillospirales|f__Oscillospiraceae|g__UCG-002</td><td>LGF</td><td>0.000809016</td><td>0.022190179</td></tr>
<tr><td>LGF - CGF</td><td>k__Bacteria|p__Firmicutes|c__Clostridia|o__Clostridiales|f__Clostridiaceae|g__Clostridium_sensu_stricto_1</td><td>LGF</td><td>0.000882119</td><td>0.022804955</td></tr>
<tr><td>LGF - CGF</td><td>k__Bacteria|p__Firmicutes|c__Clostridia|o__Christensenellales|f__Christensenellaceae|g__Christensenellaceae_R-7_group</td><td>LGF</td><td>5.83E-05</td><td>0.00689602</td></tr>
<tr><td>LGF - CGF</td><td>k__Bacteria|p__Firmicutes|c__Clostridia|o__Lachnospirales|f__Lachnospiraceae|g__Lachnospiraceae_UCG-004</td><td>CGF</td><td>0.000722548</td><td>0.020606002</td></tr>
<tr><td>LGF - CGF</td><td>k__Bacteria|p__Firmicutes|c__Clostridia|o__Oscillospirales|f__Oscillospiraceae|g__NK4A214_group</td><td>LGF</td><td>0.000559422</td><td>0.017795664</td></tr>
<tr><td>LGF - CGF</td><td>k__Bacteria|p__Firmicutes|c__Clostridia|o__Peptostreptococcales-Tissierellales|f__Anaerovoracaceae|g__Family_XIII_AD3011_group</td><td>LGF</td><td>0.001903869</td><td>0.037589199</td></tr>
<tr><td>LGF - NGF</td><td>k__Bacteria|p__Proteobacteria|c__Gammaproteobacteria|o__Enterobacterales|f__Enterobacteriaceae|g__Escherichia-Shigella</td><td>LGF</td><td>0.001600026</td><td>0.03329783</td></tr>
<tr><td>LGF - NGF</td><td>k__Bacteria|p__Proteobacteria|c__Gammaproteobacteria|o__Enterobacterales|f__Enterobacteriaceae|g__Klebsiella</td><td>LGF</td><td>0.00167648</td><td>0.03442373</td></tr>
<tr><td>LGF - NGF</td><td>k__Bacteria|p__Firmicutes|c__Negativicutes|o__Veillonellales-Selenomonadales|f__Selenomonadaceae|g__Megamonas</td><td>NGF</td><td>2.85E-05</td><td>0.004383551</td></tr>
<tr><td>LGF - NGF</td><td>k__Bacteria|p__Verrucomicrobiota|c__Verrucomicrobiae|o__Verrucomicrobiales|f__Akkermansiaceae|g__Akkermansia</td><td>LGF</td><td>0.000103683</td><td>0.007983626</td></tr>
<tr><td>LGF - NGF</td><td>k__Bacteria|p__Firmicutes|c__Clostridia|o__Lachnospirales|f__Lachnospiraceae|g__[Eubacterium]_ruminantium_group</td><td>NGF</td><td>0.000972329</td><td>0.02454731</td></tr>
<tr><td>LGF - OGF</td><td>k__Bacteria|p__Firmicutes|c__Negativicutes|o__Veillonellales-Selenomonadales|f__Selenomonadaceae|g__Megamonas</td><td>OGF</td><td>0.000269639</td><td>0.014830147</td></tr>
<tr><td>LGM - CGM</td><td>k__Bacteria|p__Firmicutes|c__Negativicutes|o__Veillonellales-Selenomonadales|f__Selenomonadaceae|g__Megamonas</td><td>CGM</td><td>0.000332332</td><td>0.01557348</td></tr>
<tr><td>LGM - CGM</td><td>k__Bacteria|p__Proteobacteria|c__Gammaproteobacteria|o__Pseudomonadales|f__Pseudomonadaceae|g__Pseudomonas</td><td>LGM</td><td>9.67E-05</td><td>0.007983626</td></tr>
<tr><td>LGM - CGM</td><td>k__Bacteria|p__Firmicutes|c__Clostridia|o__Oscillospirales|f__Oscillospiraceae|g__UCG-002</td><td>LGM</td><td>0.000541991</td><td>0.017795664</td></tr>
</table>

LGM - CGM	*k__Bacteria|p__Firmicutes|c__Clostridia|o__Lachnospirales|f__Lachnospiraceae|g__Lachnospira*	CGM	0.001047247	0.025599383
LGM - CGM	*k__Bacteria|p__Firmicutes|c__Clostridia|o__Christensenellales|f__Christensenellaceae|g__Christensenellaceae_R-7_group*	LGM	0.000283702	0.015065532
LGM - CGM	*k__Bacteria|p__Firmicutes|c__Clostridia|o__Oscillospirales|f__Oscillospiraceae|g__NK4A214_group*	LGM	0.000848612	0.022532101
LGM - CGM	*k__Bacteria|p__Firmicutes|c__Clostridia|o__Lachnospirales|f__Lachnospiraceae|g__[Eubacterium]_ruminantium_group*	LGM	0.000173394	0.0106811
LGM - CGM	*k__Bacteria|p__Firmicutes|c__Clostridia|o__Oscillospirales|f__Oscillospiraceae|g__UCG-005*	LGM	0.000434671	0.017795664
LGM - CGM	*k__Bacteria|p__Firmicutes|c__Clostridia|o__Peptostreptococcales-Tissierellales|f__Anaerovoracaceae|g__Family_XIII_AD3011_group*	LGM	0.00013129	0.009190322
LGM - NGM	*k__Bacteria|p__Verrucomicrobiota|c__Verrucomicrobiae|o__Verrucomicrobiales|f__Akkermansiaceae|g__Akkermansia*	LGM	0.002412318	0.044225822
LGM - NGM	*k__Bacteria|p__Firmicutes|c__Clostridia|o__Oscillospirales|f__Ruminococcaceae|g__UBA1819*	LGM	0.000394674	0.017365647
LGM - OGM	*k__Bacteria|p__Proteobacteria|c__Gammaproteobacteria|o__Enterobacterales|f__Enterobacteriaceae|g__Escherichia-Shigella*	LGM	0.000888505	0.022804955
LGM - OGM	*k__Bacteria|p__Firmicutes|c__Negativicutes|o__Veillonellales-Selenomonadales|f__Selenomonadaceae|g__Megamonas*	OGM	1.10E-06	0.000424889
LGM - OGM	*k__Bacteria|p__Firmicutes|c__Clostridia|o__Lachnospirales|f__Lachnospiraceae|g__Lachnospira*	OGM	0.002190094	0.041131031
LGM - OGM	*k__Bacteria|p__Firmicutes|c__Bacilli|o__Lactobacillales|f__Lactobacillaceae|g__Lactobacillus*	LGM	0.001295437	0.028098216
NGF - CGF	*k__Bacteria|p__Proteobacteria|c__Gammaproteobacteria|o__Enterobacterales|f__Enterobacteriaceae|g__Escherichia-Shigella*	CGF	0.002675546	0.047910933
NGF - CGF	*k__Bacteria|p__Proteobacteria|c__Gammaproteobacteria|o__Pseudomonadales|f__Pseudomonadaceae|g__Pseudomonas*	NGF	9.01E-07	0.000424889
NGF - CGF	*k__Bacteria|p__Firmicutes|c__Clostridia|o__Lachnospirales|f__Lachnospiraceae|g__[Eubacterium]_ruminantium_group*	NGF	7.49E-05	0.007694355
NGM - CGM	*k__Bacteria|p__Firmicutes|c__Clostridia|o__Lachnospirales|f__Lachnospiraceae|g__[Eubacterium]_ruminantium_group*	NGM	4.22E-05	0.005907397
OGF - CGF	*k__Bacteria|p__Proteobacteria|c__Gammaproteobacteria|o__Pseudomonadales|f__Pseudomonadaceae|g__Pseudomonas*	OGF	1.02E-05	0.002577538
OGF - CGF	*k__Bacteria|p__Firmicutes|c__Clostridia|o__Lachnospirales|f__Lachnospiraceae|g__Dorea*	OGF	0.001289323	0.028098216
OGF - CGF	*k__Bacteria|p__Firmicutes|c__Clostridia|o__Peptostreptococcales-Tissierellales|f__Peptostreptococcaceae|g__Terrisporobacter*	OGF	0.000475231	0.017795664
OGM - CGM	*k__Bacteria|p__Firmicutes|c__Clostridia|o__Oscillospirales|f__Ruminococcaceae|g__Subdoligranulum*	OGM	0.000821325	0.022190179
OGM - CGM	*k__Bacteria|p__Proteobacteria|c__Gammaproteobacteria|o__Pseudomonadales|f__Pseudomonadaceae|g__Pseudomonas*	OGM	1.51E-05	0.002577538
OGM - CGM	*k__Bacteria|p__Firmicutes|c__Clostridia|o__Lachnospirales|f__Lachnospiraceae|g__[Eubacterium]_ruminantium_group*	OGM	0.000150174	0.009907657
OGM - CGM	*k__Bacteria|p__Firmicutes|c__Clostridia|o__Oscillospirales|f__Ruminococcaceae|g__Negativibacillus*	OGM	0.001103251	0.02654697
LB - HB	*k__Bacteria|p__Firmicutes|c__Negativicutes|o__Veillonellales-Selenomonadales|f__Selenomonadaceae|g__Megamonas*	HB	0.000120419	0.019869068
LB - HB	*k__Bacteria|p__Verrucomicrobiota|c__Verrucomicrobiae|o__Verrucomicrobiales|f__Akkermansiaceae|g__Akkermansia*	LB	0.001226991	0.029671779
LB - HB	*k__Bacteria|p__Firmicutes|c__Clostridia|o__Oscillospirales|f__Oscillospiraceae|g__UCG-002*	LB	0.00191124	0.035390215
LB - HB	*k__Bacteria|p__Firmicutes|c__Clostridia|o__Lachnospirales|f__Lachnospiraceae|g__Lachnospira*	HB	0.000920821	0.029671779
LB - HB	*k__Bacteria|p__Actinobacteriota|c__Coriobacteriia|o__Coriobacteriales|f__Coriobacteriaceae|g__Collinsella*	LB	0.002721637	0.044907003
LB - HB	*k__Bacteria|p__Firmicutes|c__Clostridia|o__Christensenellales|f__Christensenellaceae|g__Christensenellaceae_R-7_group*	LB	0.000718065	0.029620192
LB - HB	*k__Bacteria|p__Firmicutes|c__Clostridia|o__Oscillospirales|f__Oscillospiraceae|g__UCG-005*	LB	0.000497157	0.027343641
LB - HB	*k__Bacteria|p__Firmicutes|c__Clostridia|o__Peptostreptococcales-Tissierellales|f__Anaerovoracaceae|g__Family_XIII_AD3011_group*	LB	0.00036166	0.027343641
LB - MB	*k__Bacteria|p__Verrucomicrobiota|c__Verrucomicrobiae|o__Verrucomicrobiales|f__Akkermansiaceae|g__Akkermansia*	LB	0.001930375	0.035390215
LB - MB	*k__Bacteria|p__Firmicutes|c__Clostridia|o__Oscillospirales|f__Oscillospiraceae|g__UCG-005*	LB	0.001258803	0.029671779
